# Case Report: Selexipag in pediatric pulmonary hypertension: Initiation, transition, and titration

**DOI:** 10.3389/fped.2023.1050508

**Published:** 2023-03-08

**Authors:** Jenna M. Faircloth, Neelam D. Bhatt, Corey A. Chartan, Ryan D. Coleman, Natalie Villafranco, Fadel E. Ruiz, Raysa Morales-Demori, Elise Whalen, Erin Ely, Rozmeen Fombin, Nidhy P. Varghese

**Affiliations:** ^1^ Department of Pharmacy, Texas Children’s Hospital, Houston, TX, United States; ^2^ Department of Pediatrics, Division of Critical Care and Pulmonology, Baylor College of Medicine, Texas Children’s Hospital, Houston, TX, United States; ^3^ Department of Pediatrics, Division of Pulmonology, Baylor College of Medicine, Texas Children’s Hospital, Houston, TX, United States; ^4^ Department of Pediatrics, Division of Critical Care, Baylor College of Medicine, Texas Children’s Hospital, Houston, TX, United States; ^5^ Department of Pulmonary Medicine, Texas Children’s Hospital, Houston, TX, United States

**Keywords:** pediatric pulmonary hypertension, selexipag, treprostinil, initiation, transition, prostacyclin

## Abstract

Selexipag, a selective prostacyclin receptor agonist, is approved for treating pulmonary arterial hypertension in WHO Group 1 adult patients. Compared to parenteral prostacyclin formulations, selexipag offers a significant improvement in patient’s and caregiver’s quality of life because of its oral formulation, frequency of administration, and mechanism of action. Although experience in the pediatric population is limited and selexipag is not FDA-approved for use in the pediatric pulmonary hypertension population, many US pediatric centers are expanding the use of this therapy to this younger population. We report our institution's experience in the use of selexipag to treat pulmonary hypertension in children under 10 years of age, between 10 and 30 kg. Seven patients were initiated on selexipag therapy including *de novo* initiation and transition from intravenous treprostinil to oral selexipag. All patients were on stable background therapy with phosphodiesterase-5 inhibitor and endothelin receptor antagonist therapies at baseline. All patients reached their planned goal selexipag dose during admission without the need for changes to the titration schedule and without hemodynamic deterioration. In our experience, oral selexipag is safe and well-tolerated in young pediatric patients with pulmonary hypertension. Based on our favorable experience, we developed an institution-specific selexipag process algorithm for continued successful use in the pediatric population.

## 1. Introduction

Pediatric pulmonary arterial hypertension (PH) is a rare, progressive disease associated with significant morbidity and mortality. Current therapies target three main physiologic pathways: the nitric oxide, endothelin, and prostacyclin pathways. Oral agents approved by the U.S. Food and Drug Administration (FDA) for the treatment of chronic PH in adults include phosphodiesterase-5 inhibitors (sildenafil, tadalafil), endothelin receptor antagonists (bosentan, ambrisentan, macitentan), prostacyclin analogs such as oral treprostinil, and soluble guanylate cyclase stimulators (riociguat). Oral agents are generally preferred by patients and families over parenteral or inhaled therapies due to lower treatment burden and are therefore associated with increased medication adherence. Selexipag, a selective prostacyclin receptor agonist, was approved by the FDA in 2015 as the second oral prostacyclin pathway-targeted therapy option for adult WHO Group 1 patients. Clinical trials demonstrated a lower incidence of side effects, less frequent dosing, and administration without regard to food compared to oral treprostinil (1). The longer half-life successfully maintained efficacy and reduced side effects in the setting of a missed dose. As with oral treprostinil, avoiding subcutaneous (SQ) or intravenous (IV) access was associated with improved quality of life.

SQ administration of prostacyclins is associated with pain at the SQ site, and central line infections are a common complication with chronic IV access. Central/SQ line care, dressing changes, flushes, travel restrictions and precautions, and limited ability to participate in similar activities as their peers often make parenteral therapy complex and time-consuming. The burden parenteral prostacyclin therapy places on patients, their caregivers, and their families is significant and widespread. Oral selexipag has offered flexibility to caregivers and reduced complications to patients for those who can tolerate it. Response to selexipag therapy and clinical stability are similar to those seen with parenteral prostacyclin in adult studies (2, 3). Given these advantages and pharmacokinetics, selexipag is an attractive therapy choice. Therefore, although experience in larger pediatric populations is generally limited and selexipag is not FDA-approved for use in pediatric patients, many pediatric centers in the United States are expanding the use of this therapy to children with reported success (4–8).

However, the safety and efficacy of selexipag have not been established in pediatric patients, nor is selexipag FDA-approved for pediatric use. At the time of manuscript preparation, there is an ongoing randomized, double-blind, placebo-controlled clinical trial of selexipag in pediatric PH patients aged 2–17 years evaluating time to disease progression, with estimated study completion in 2028 (9). As trial results are awaited, it is essential that large center experience is shared to assist in current clinical practice and to identify optimal initiation, transition, and titration of selexipag therapy. There are adult data that can be extrapolated to guide clinical practice; however, pharmacokinetics vary greatly between these two populations; therefore, the framework for selexipag use in pediatrics has to be tailored to this population. Therefore, in this case series, we present our experience in rapid selexipag initiation and transition from parenteral treprostinil in young pediatric PH patients, along with an institution-specific process algorithm, to bridge the gap resulting from the lack of a standardized approach in this population.

## 2. Methodology

**Figure 1:**
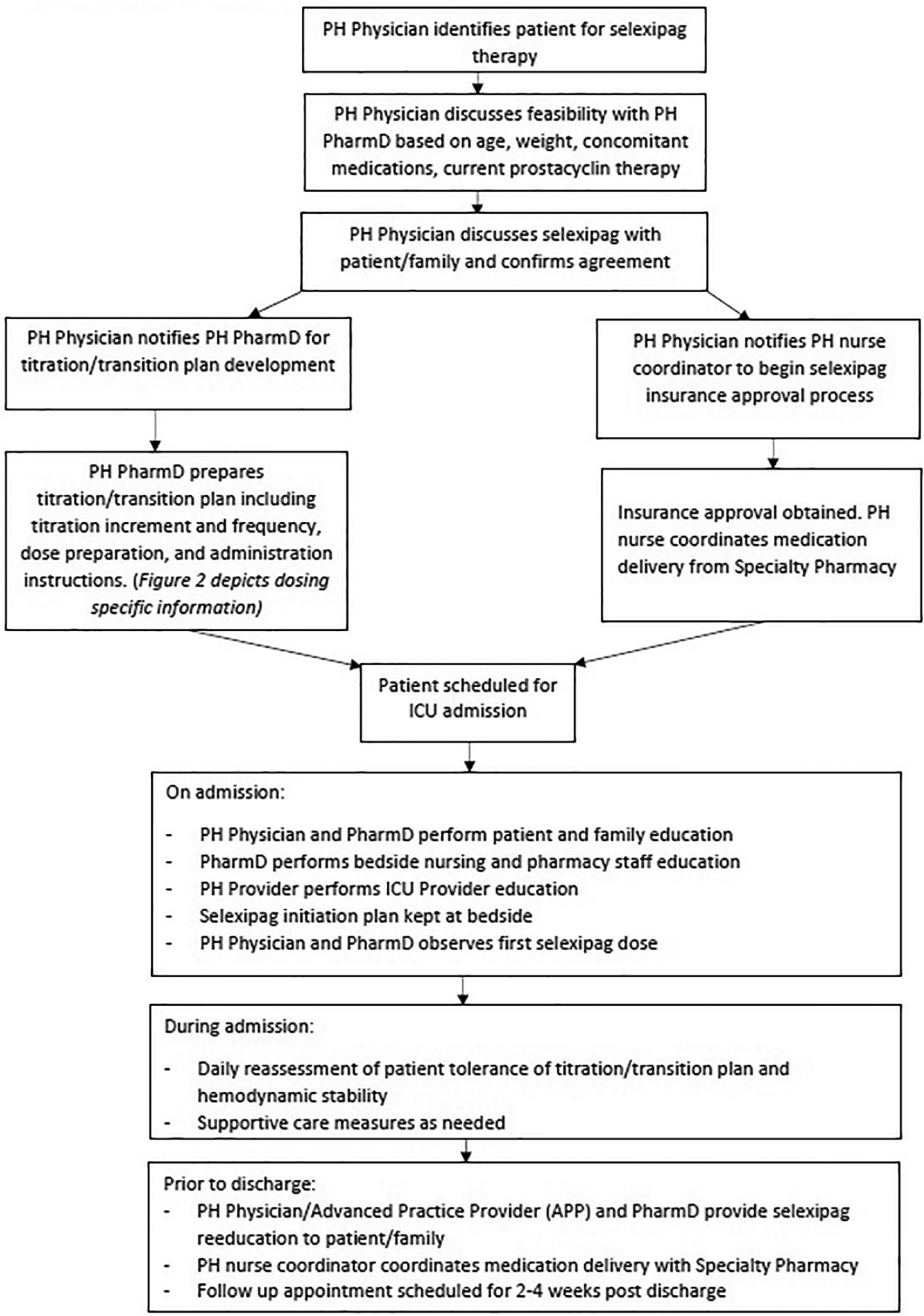
Institution-specific selexipag initiation algorithm.

Seven patients were initiated on selexipag therapy. A standardized process was developed for the initiation and rapid transition to selexipag (Figure 1). Once candidates for selexipag initiation or transition were identified by the treating physician, medication approval was obtained. The patient was then scheduled for admission to the pediatric intensive care unit (PICU) to begin selexipag in a monitored setting.

Upon admission, throughout the hospital stay, and particularly at discharge, the PH pharmacist and PH physician educated the patient, family, and bedside nursing staff regarding possible adverse effects of titration (hypotension, chest pain, dyspnea, hypoxemia). Using pictorial graphs and medication calendars, education was provided on different tablet strengths, the importance of adherence, and timely administration.

For *de novo* initiation in children <30 kg, selexipag was initiated at 50 μg every 12 h and rapidly titrated up in 50 μg per dose per day increments until the goal dose was achieved, roughly 4 days. To determine the goal dose, we utilized an equivalent of IV treprostinil; 10 ng/kg/min IV treprostinil was equivalent to 100 μg selexipag every 12 h for patients <30 kg and 10 ng/kg/min IV treprostinil was equivalent to 200 μg selexipag every 12 h for patients ≥30 kg. Upon reaching the goal dose, the patient was discharged to continue slower titration in the outpatient setting.

Similarly, to determine selexipag goal dose and titration increments for transitions from IV treprostinil to oral selexipag, we utilized an equivalent of IV treprostinil; 10 ng/kg/min IV treprostinil was equivalent to 100 mcg selexipag every 12 h for patients <30 kg and 10 ng/kg/min IV treprostinil was equivalent to 200 μg selexipag every 12 h for patients ≥30 kg. During the rapid transitions from IV treprostinil, selexipag was administered first and immediately followed by IV treprostinil dose reduction by an increment of 2–5 ng/kg/min per step. Selexipag 200 μg tablets were used to facilitate dose titrations and adjustments. For smaller-dose titration steps and/or patients who could not swallow tablets, 200 μg tablets were dissolved in 4 ml of water to yield a mixture of 50 μg/ml, as described by Koo et al. (4). Dose titrations were directly observed by the inpatient PH team, and every patient was monitored as per PICU protocol.

We present a review of the seven patients who received selexipag therapy from May 2020 to September 2021 at our institution. Each patient case was reviewed for baseline information: age, WHO Group classification, comorbidities, background therapy, weight at the time of selexipag treatment initiation, 6-min walk distance (6 MWD), right ventricular (RV) pressure estimate, and baseline mean pulmonary artery pressure (mPAP) as measured within the past year.

## 3. Cases

### 3.1. *De novo* selexipag initiation and titration

#### 3.1.1. Case 1

A 3-year-old boy with WHO Group 1 PAH presented with the worsening disease despite dual-targeted therapy. The mPAP at diagnosis was 36 mmHg. However, 2 years later, when referred to our center, the RV systolic pressure estimate by echocardiogram was at least 61 mmHg. Past medical history was significant for persistent pulmonary hypertension of the newborn and bone marrow transplant in the context of MECOM mutation. Parenteral prostacyclin therapy was recommended but declined by the family due to the perceived treatment burden. Selexipag therapy was therefore agreed upon as an alternative.

At the time of selexipag initiation, the patient weighed 14.6 kg. A baseline 6-min walk test could not be done due to age/developmental status. The baseline echocardiogram estimate of RV pressure was at least 70 mmHg. Cardiac catheterization had not been repeated since diagnosis 2 years prior. Selexipag was initiated at 50 μg (1 ml of 200 μg/4 ml solution) every 12 h. Over the course of the next 4 days, selexipag dosing was titrated by 50 μg per dose once daily up to the goal dose of 200 μg every 12 h. The patient tolerated the transition well without significant events. Six months after initiation, the family reported subjective improvement in activity and endurance. On serial assessments, the child was noted to have increased weight gain and a consistent increase in 6MWD. The echocardiographic estimate of RV systolic pressure was 77 mmHg. Although he was clinically progressing in a positive direction, since his echocardiogram was largely unchanged, the selexipag dose was increased without complication by 100 μg per dose weekly up to 400 μg every 12 h. Functional class improved from class III to class II. Cardiac catheterization was performed 19 months after selexipag initiation, and mean PAP was 56 mmHg, consistent with echocardiograms at that time. Concerns about consistent oral dosing administration and absorption prompted reconsideration of parenteral prostacyclin therapy, so the child was ultimately transitioned successfully from selexipag to IV treprostinil.

#### 3.1.2. Case 2

A 5-year-old girl with trisomy 21 and PH associated with the late repair of atrioventricular septal defect (AVSD) presented with worsening PH. Comorbidities included a history of extracorporeal membrane oxygenation, tracheostomy, gastrostomy tube placement, adrenal insufficiency, factor V Leiden deficiency, and hypothyroidism. Although she had been classified as a Group 1 PAH patient, recurrent aspiration and associated chronic lung disease were suspected, prompting consideration for Group 3 exacerbating diseases. She had been on dual therapy; however, interval catheterization showed elevated PA pressures and indexed pulmonary vascular resistance. Selexipag was selected due to limited caregiver resources to support parenteral prostacyclin therapy in the outpatient setting.

At the time of selexipag initiation, the patient weighed 15.1 kg. The baseline 6-min walk distance was 254 m. The baseline echocardiogram estimate of RV pressure was 44 mmHg. Baseline cardiac catheterization was 53 mmHg before selexipag initiation. Selexipag was initiated at a dose of 50 μg (1 ml of 200 μg/4 ml solution) every 12 h over 4 days. Selexipag dose was titrated by 50 μg per dose each day up to the goal dose of 200 μg every 12 h. Once outpatient, dose titration continued to 250 μg every 12 h. However, with this continued dose titration, the caregiver noted facial flushing, decreased appetite, intermittent diarrhea, and headache, so the dose was decreased back to selexipag 200 μg every 12 h. Six months after initiation, the child's functional status was judged to be improved to class I, and the family reported subjective improvement in activity and endurance. Her 6MWD was improved to 314 m. The echocardiographic estimate of RV systolic pressure was 43 mmHg. Interval cardiac catheterization had not yet been completed (elapsed time since initiation = 12 months) at the time of manuscript submission.

### 3.2. Parenteral treprostinil to selexipag transition and titration

#### 3.2.1. Case 3

A 5-year-old girl on triple combination therapy to treat severe hereditary PAH (Group 1 secondary to BMPR2+ and KCNA5+ mutations) was evaluated for conversion from IV treprostinil to selexipag due to repeated admissions for central venous line-related complications (damaged line and infections).

At the time of selexipag initiation, the patient weighed 14 kg. The baseline 6-min walk distance was 190 m. The baseline echocardiogram could not estimate RV pressure; however, the septal motion was moderately to severely flattened with associated moderate RV dysfunction. Baseline cardiac catheterization measured mPAP 59 mmHg before selexipag initiation. The patient was started on selexipag 100 μg every 12 h and increased by 100 μg per dose daily while decreasing IV treprostinil by 5 ng/kg/min every 12 h. The transition from IV treprostinil 62 ng/kg/min to selexipag 600 μg every 12 h was completed in 6 days. The transition was well tolerated. Six months after initiation, the child's functional status was judged to be class II. The family reported subjective improvement, and at the time of this study, she has not had any more admissions since conversion to selexipag. Her 6MWD was 292 m at 6 months post-transition. The echocardiographic estimate of RV systolic pressure could not be quantified; however, the septum was noted to be flattened, and RV systolic function was judged normal. Interval cardiac catheterization has not been done due to social barriers affecting scheduling (elapsed time since transition = 23 months).

#### 3.2.2. Case 4

An 8-year-old girl was newly diagnosed with Group 1 PAH after 1 year of worsening dyspnea with exercise, syncopal episodes, and severe right ventricular dysfunction necessitating extracorporeal membrane oxygenation support. Baseline cardiac catheterization data were obtained while on (clamped) extracorporeal support ad epinephrine, vasopressin, and calcium chloride infusions. The mean PA pressure on this support was measured to be 35 mmHg. She was able to come off mechanical support on upfront combination therapy of tadalafil, ambrisentan, and IV treprostinil 36 ng/kg/min. The patient had a surprisingly robust response to therapy, with complete normalization of the echocardiogram. Due to limited resources for the family and the normal echocardiogram, prostacyclin therapy was adjusted from IV treprostinil to selexipag.

At the time of selexipag transition, the patient weighed 30.6 kg. Baseline 6MWD could not be done since the child was still admitted to intensive care and was judged to be too critically ill to participate in testing. A baseline echocardiogram performed on IV treprostinil prior to transition could not quantify the RV pressure estimate; however, septal motion and RV function were normal. She was started on selexipag 100 μg every 12 h. IV treprostinil was decreased by 4 ng/kg/min twice daily immediately after each selexipag dose. Selexipag was increased by 100 μg per dose daily in the morning until a goal dose of 800 μg every 12 h was achieved. She transitioned over 6 days and tolerated the transition well with no side effects. At the 6-month follow-up, the child's functional status was judged to be class I. Her 6MWD at 6 months was 469 m. The echocardiographic estimate of RV systolic pressure was 33 mmHg. Interval cardiac catheterization showed an mPAP of 30 mmHg.

#### 3.2.3. Case 5

An 8-year-old boy with idiopathic PH (due to ABCA3 point mutation), WHO Group 1, on triple therapy with ambrisentan, tadalafil, and SQ treprostinil dose of 100 ng/kg/min was identified for selexipag therapy to improve quality of life after 6.5 years of SQ therapy. At the time of selexipag initiation, the patient weighed 18 kg. The baseline 6-min walk distance was 391 m. The baseline echocardiogram estimate of RV pressure was 64 mmHg. Baseline cardiac catheterization, done on triple therapy 2 years before transition, measured an mPAP of 26 mmHg. Of note, it had been 35 mmHg on diagnostic catheterization 5 years prior. Selexipag was initiated at 100 mcg every 12 h. Intravenous treprostinil was decreased by 5 ng/kg/min twice daily immediately after every selexipag dose. Selexipag was increased by 100 mcg per dose once daily up to a goal of 600 μg every 12 h over 6 days. The patient tolerated the transition well during the admission. He was discharged 12 h after the last dose change without any adverse events. Selexipag dose was increased outpatient to 1,200 μg every 12 h over the following year. Six months after initiation, the child's functional status was still considered class I, and the family reported great energy levels as demonstrated by the child running laps with other children. His 6 MWD was 566 m. The echocardiographic estimate of RV systolic pressure was 52 mmHg. Interval cardiac catheterization 9 months after transition showed an mPAP of 29 mmHg.

#### 3.2.4. Case 6

A 10-year-old boy with PH, WHO Group 1, on triple therapy with ambrisentan, tadalafil, and IV treprostinil 128 ng/kg/min, was identified for selexipag therapy. The treatment plan was tailored to encourage remodeling for the ultimate closure of a patent ductus arteriosus (PDA); however, cardiac catheterization data did not support intervention. Unfortunately, lung transplantation was not a good option because of the preserved right ventricular function. Therefore, in consideration for quality of life given the unlikely consideration for ductal closure, conversion from IV treprostinil to selexipag was offered.

At the time of selexipag initiation, the patient weighed 23 kg. His baseline 6MWD was 547 m. The baseline echocardiogram showed a bidirectional shunt across the PDA. Baseline cardiac catheterization measured an mPAP of 52 mmHg before selexipag transition. He was on IV treprostinil 128 ng/kg/min, which was weaned down weekly outpatient to 100 ng/kg/min, with regular echocardiographic monitoring. The reason to decrease the treprostinil dose outpatient prior to transitioning to selexipag was to decrease overall hospital stay and start selexipag at a dose that fits within the approved adult dosing recommendation (i.e., <1,600 μg twice daily.) When he reached the target IV treprostinil dose of 100 ng/kg/min, he was admitted and started on selexipag 100 μg every 12 h. Treprostinil was decreased by 5 ng/kg/min twice daily immediately after every selexipag dose. Selexipag was increased by 100 mcg per dose once daily up to a target dose of 1,000 μg every 12 h over 10 days. He was discharged 24 h after the last dose change. He had no side effects during admission, and his upper and lower extremity oxygen saturations were noted to be matched. Selexipag was increased outpatient to 1,200 μg every 12 h to address frequent leg cramps and tiring; however, he had increased side effects (nausea, stomach pain, and headache) with a morning dose of selexipag. This was treated with more consistent meals, ondansetron pretreatment, and oxygen supplementation during meals. As side effects improved, his dose was increased over 2 months to 1,600 μg every 12 h outpatient. After reaching the maximum dose, he reported a good energy level and fewer foot desaturations. Six months after initiation, the child's functional status was class II. His 6MWD was 577 m. Echocardiograms continued to demonstrate the bidirectional shunt (unchanged) at the PDA. Interval cardiac catheterization showed an mPAP of 51 mmHg.

#### 3.2.5. Case 7

A 2-year-old boy patient with repaired D-transposition of the great arteries who was subsequently palliated with reverse Potts shunt procedure for continued severe disease presented for conversion from SQ treprostinil to selexipag in the post-Potts shunt period. The postoperative period was unremarkable; however, the child experienced frequent site changes and skin reactions to the subcutaneous dressing, so he was transitioned to selexipag. At the time of selexipag initiation, the patient weighed 10.5 kg. The baseline 6-min walk distance could not be done due to age and mobility. The baseline echocardiogram showed a bidirectional shunt at the level of the reverse Potts shunt. Baseline cardiac catheterization measured an mPAP of 76 mmHg before selexipag titration. The patient started titration at a dose of 50 μg selexipag (1 ml of 200 μg/4 ml solution) every 12 h. In contrast to the other treprostinil to selexipag transition patients, 2 ng/kg/min treprostinil steps were selected due to the patient's previous history of sensitivity with dose escalation of prostacyclin, during SQ treprostinil initiation. Treprostinil was decreased by 2 ng/kg/min twice daily immediately after every selexipag dose. Selexipag was increased by 50 mcg per dose once daily to a goal dose of 200 mcg every 12 h over 5 days. He tolerated the transition well, with no side effects. He was discharged 12 h after the last dose change. As an outpatient, he was transitioned to tadalafil, and the selexipag dose was increased to 600 mcg every 12 h over 4 months. Six months after initiation, the child's functional status continued to be class I. The family reported matched upper and lower extremity saturations. The child was noted to have consistent weight gain. At the time of this study, he remained developmentally too young for 6MWD. Echocardiograms were stable on selexipag, demonstrating the bidirectional shunt across the reverse Potts shunt. Interval cardiac catheterization was not sought post-transition (elapsed time = 15 months).

### 3.3. Clinical characteristics of patients

Patient demographics prior to selexipag therapy are displayed in Table 1. Seven patients were initiated on selexipag therapy. Two (29%) patients had *de novo* initiation, and five (71%) patients were transitioned on admission from intravenous treprostinil to oral selexipag. Four of seven (57%) patients were boys. Four of seven (57%) patients were 5 years old or younger. All patients were WHO Group 1 and were on stable background therapy with a PDE5 inhibitor and endothelin receptor antagonist. Selexipag dosing strategies and clinical characteristics before and after selexipag initiation are also represented in Table 1.

**Table 1:** Characteristics of selexipag initiation patients.

Pt Case ID	WHO group	Age at initiation (years)	Gender (M/F)	Weight at initiation (kg)	Indications for SEL initiation	Concomitant medications	IV TRE dose at SEL initiation	SEL dose at initiation	SEL dose at hospital discharge	Initiation duration (d)	NYHA FC (pre/post)	6 MWD (m) (pre/post)	BNP ng/m (pre/post)	Mean PAP (mmHg) (pre/post)	PVRi (WUm2) (pre/post)	Trans pulmonary gradient (mmHg) (pre/post)
*De novo* initiation																
1	1	3	M	14.6	Family refusal of parenteral therapy	Tadalafil, ambrisentan	NA	50 μg BID	200 μg BID	4	III/II	NA/382.5	134/49.7	36/56	8.74/12.6	NA/44
2	1, 3	5	F	15.1	Psychosocial; lack of resources for parenteral prostacyclins	Tadalafil, ambrisentan	NA	50 μg BID	200 μg BID	4	I/I	254.14/314.12	41.3/55	53/NA	11.2/NA	43/NA
Parenteral treprostinil to oral selexipag																
3	1	5	F	14	Central line concerns/frequent admissions for line infections	Tadalafil, ambrisentan IV TRE	62 ng/kg/min	100 μg BID	600 μg BID	6	II/I	189.3/291	54.6/<10	59/NA	16.9/NA	47/NA
4	1	8	F	30.6	Psychosocial; lack of parenteral prostacyclin resources in home city/state	Tadalafil, ambrisentan, IV TRE	36 ng/kg/min	100 μg BID	800 μg BID	6	IV/I	NA/469.25	45/11	35/NA	8.9/NA	22/NA
5	1, 5	8	M	18	Quality of life	Tadalafil, ambrisentan, SQ TRE	60 ng/kg/min	100 μg BID	600 μg BID	6	II/II	566/600	<10/5	26/29	2.2/3.1	11/14
6	1, 3	10	M	23	Compassion-ate conversion from central line to oral therapy to improve quality of life	Tadalafil, ambrisentan, IV TRE	100 ng/kg/min	100 μg BID	1,000 μg BID	10	II/III	547/576.6	62/N/A	51/51	7.4/9.9	37/43
7	1, 3	2	F	10.5	Central line concerns	Sildenafil, bosentan, SQ TRE	20 ng/kg/min	50 μg BID	200 μg BID	5	III/II	NA	53/110	76/NA	18.8/NA	63/NA

TRE, treprostinil; SEL, selexipag; BID, twice daily, spaced by 12 h; IV, intravenous; 6 MWD, 6-min walk distance; PAP, pulmonary artery pressure.

## 4. Discussion

**Figure 2:**
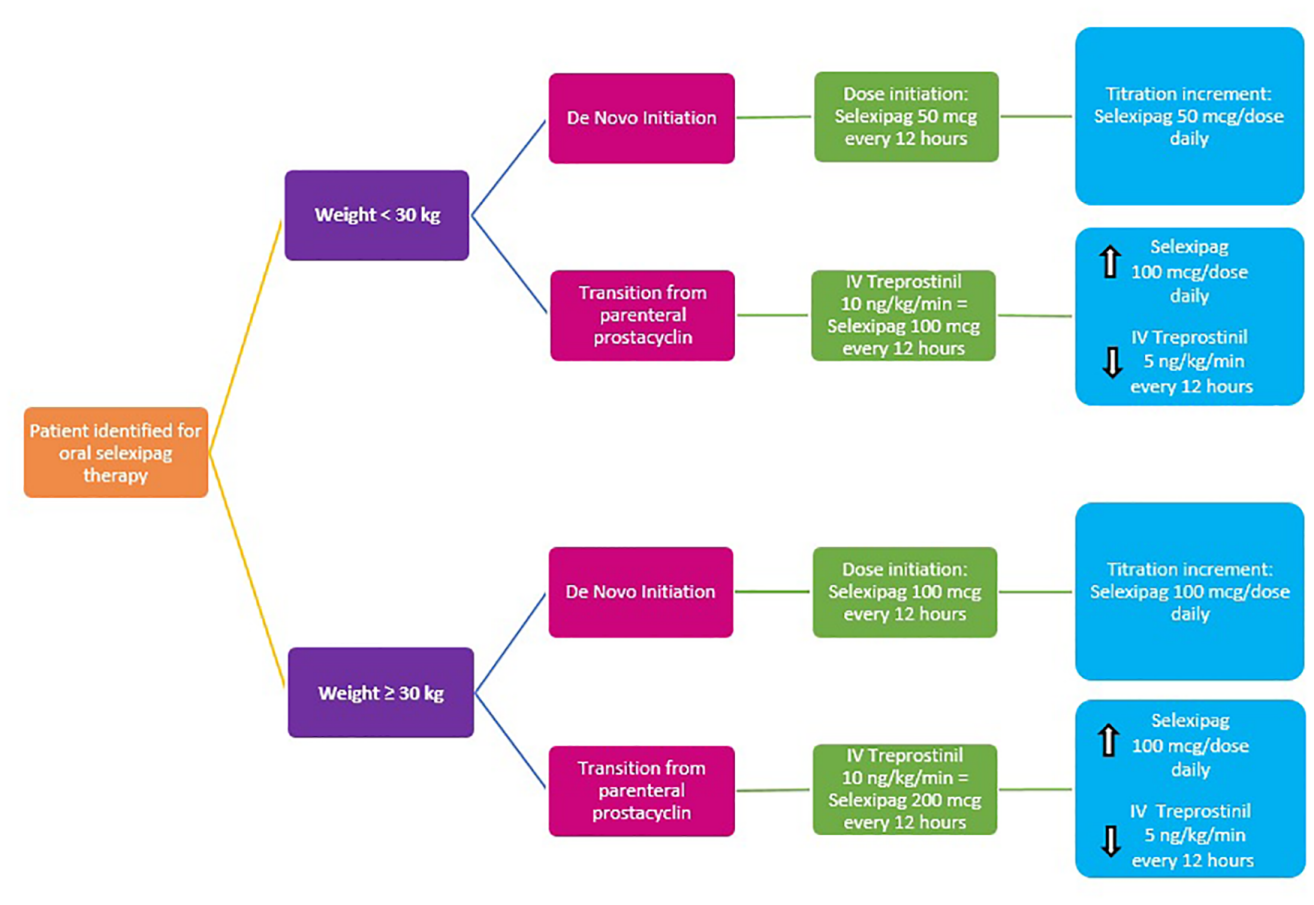
Institution-specific selexipag initiation dosing guide.

This case series describes our experience with *de novo* initiation and rapid transition from treprostinil to oral selexipag in young pediatric patients aged 2–10 years old. Our experience allowed us to develop an institution-specific algorithm (Figure 1) and selexipag dosing manual (Figure 2) to help guide our practice in using selexipag in younger patients.

The dosing strategy and transition methodology were developed on principles of dose escalation of parenteral prostacyclin therapy, treprostinil and selexipag pharmacokinetics, and historical experience with transitions from IV to oral treprostinil. The median duration of *de novo* selexipag initiation was 4 days. The median duration of the rapid transition to selexipag was 6 days (5–10 days) as it was dependent on the patient's initial treprostinil dose and goal selexipag dose. Patients were generally ready for discharge by 12 h after receiving the final titration dose and were either discharged the same day or the next day, depending on consideration of hour of the day. All patients in our cohort completed the transition as planned, without unexpected events, adverse side effects, and any deterioration in hemodynamic parameters.

Clinical data to support the nasogastric or gastric feeding tube administration route of selexipag is lacking in this population; however, it was deemed successful based on subjective and objective improvement, supporting absorption, in both of our patients. We used the preparation method described in the 2021 case series by Koo et al. (4) with dosing *via* a nasogastric or gastric feeding tube.

Factors we considered relevant for pediatric selexipag therapy were stable disease (i.e., without worse PH symptoms or disease progression), stable prostacyclin doses, quality of life, and availability of resources to support parenteral prostacyclin therapy. Prior to selexipag, our patients were challenged by SQ site infections, SQ site pain, and central line complications. After the transition to selexipag, patients have remained hemodynamically stable and without disease progression, as documented by echo and 6MWD. We believe selexipag may play a role in reducing overall healthcare costs through reduction in unscheduled admissions as our patients admitted frequently for complications due to IV/SQ treprostinil therapy have not had an unscheduled admission since selexipag. All of our patients and their caregivers reported improvements in quality of life. We suggest that our institution-specific algorithm (Figure 1) and dosing guide (Figure 2) are new tools that can be used for safe selexipag therapy initiation in pediatric patients with stable disease.

A limitation of our case series is the size of the population. However, while our overall cohort was small, we described a consistent experience in using selexipag across a wide age and weight range. Since September 2021, we have applied this process to 10 more patients, all with the same level of success. It must also be noted that process implementation started in May 2020, during the COVID-19 global pandemic. This impacted our ability to directly monitor these patients closely for follow-up postinitiation in the outpatient setting. Due to clinical and access constraints, there were decreased encounters for in-clinic visits, 6MWD testing, echocardiography, and cardiac catheterization testing. Therefore, in some instances, follow-up may not be truly long-term or complete.

## 5. Conclusion

This case series summarizes our center's experience in introducing selexipag therapy in young pediatric patients. Using a process specific to pediatric pharmacokinetics and pharmacodynamics, *de novo* initiation and rapid transition from parenteral treprostinil to selexipag were well-tolerated and safe for children 2–10 years of age and 10–30 kg of body weight. With the support of continuous monitoring in the PICU and thorough education provided by the PH team, medical providers were able to effectively and confidently follow the titrations as planned, and each patient was successfully transitioned home to continue therapy. Follow-up data on selexipag were stable for every patient, without any evidence of clinical worsening. Given this favorable experience and follow-up, selexipag may be a reasonable oral therapy to consider in treating severe but stable pediatric PH. The process algorithm presented can be regarded as a pediatric-specific framework for successful selexipag initiation or transition in this population.

## Data Availability

The original contributions presented in the study are included in the article/Supplementary Material, further inquiries can be directed to the corresponding authors.
