# Corrigendum: Case report: Selexipag in pediatric pulmonary hypertension: initiation, transition, and titration

**DOI:** 10.3389/fped.2023.1275389

**Published:** 2023-08-21

**Authors:** Jenna M. Faircloth, Neelam D. Bhatt, Corey A. Chartan, Ryan D. Coleman, Natalie Villafranco, Fadel E. Ruiz, Raysa Morales-Demori, Elise Whalen, Erin Ely, Rozmeen Fombin, Nidhy P. Varghese

**Affiliations:** ^1^ Department of Pharmacy, Texas Children’s Hospital, Houston, TX, United States; ^2^ Department of Pediatrics, Division of Critical Care and Pulmonology, Baylor College of Medicine, Texas Children’s Hospital, Houston, TX, United States; ^3^ Department of Pediatrics, Division of Pulmonology, Baylor College of Medicine, Texas Children’s Hospital, Houston, TX, United States; ^4^ Department of Pediatrics, Division of Critical Care, Baylor College of Medicine, Texas Children’s Hospital, Houston, TX, United States; ^5^ Department of Pulmonary Medicine, Texas Children’s Hospital, Houston, TX, United States

**Keywords:** pediatric pulmonary hypertension, selexipag, treprostinil, initiation, transition, prostacyclin

A Corrigendum on Case Report: Selexipag in pediatric pulmonary hypertension: initiation, transition, and titration By Faircloth JM, Bhatt ND, Chartan CA, Coleman RD, Villafranco N, Ruiz FE, Morales-Demori R, Whalen E, Ely E, Fombin R, Varghese NP. (2023) Front Pediatr. 11:1050508. doi: 10.3389/fped.2023.1050508

In the published article, there was an error. The text of the article is corrected to reflect the most current pediatric selexipag data available at time of publication. This original statement in the abstract was from when the center-specific selexipag process was developed and implemented (April 2020). However by the time of publication (2023), more robust data on selexipag use was available and the manuscript is now corrected to reflect this.

A correction has been made to the **Abstract** section. The sentence previously stated:

“Although experience in the pediatric population is limited to case reports in older adolescent patients and selexipag is not approved for use in the pediatric pulmonary hypertension population, many pediatric centers are expanding the use of this therapy to this population.”

The corrected sentence appears below:

“Although experience in the pediatric population is generally limited and selexipag is not approved for use in the pediatric pulmonary hypertension population, many pediatric centers are expanding the use of this therapy to the younger population.”

A correction has been made to the **Introduction** section, paragraph two. The sentence previously stated:

“Therefore, although experience in the pediatric population is limited primarily to case reports in adolescent patients and selexipag is not approved for use in the pediatric pulmonary hypertension population, many pediatric centers are expanding the use of this therapy to this population (4–8).”

The corrected sentence appears below:

“Despite the lack of randomized clinical trials in pediatrics, an increasing number of centers are reporting the successful use of selexipag through case reports, series and observational studies (4–8).”

The authors apologize for this error and state that this does not change the scientific conclusions of the article in any way. The original article has been updated.

